# The efficacy of acupuncture treatment for fibromyalgia syndrome: a systematic review and meta-analysis

**DOI:** 10.3389/fmed.2025.1710642

**Published:** 2026-01-16

**Authors:** Zhengfeng Ye, Chonghong Xue, Qirun Liu, Xingyi Li, Tianqi Yu, Hewei Wei

**Affiliations:** 1 Guangzhou University of Chinese Medicine, Guangzhou, China; 2 The Third Clinical College of Guangzhou University of Chinese Medicine, Guangzhou, China; 3 The Third Affiliated Hospital of Guangzhou University of Chinese Medicine, Guangzhou, China; 4 Guangdong Research Institute for Orthopedics and Traumatology of Chinese Medicine, Guangzhou, China

**Keywords:** acupuncture, fibromyalgia syndrome, FSM, meta-analysis, systematic review

## Abstract

**Background:**

Fibromyalgia syndrome (FMS) is a common condition in rheumatology that affects patients' physical and mental health. Some studies have demonstrated the efficacy of acupuncture in alleviating pain in patients with FMS, but there is still insufficient evidence to support the improvement of pain and associated symptoms in FMS patients through acupuncture. Therefore, this study investigates whether acupuncture has therapeutic effects on patients with FMS.

**Methods:**

We searched 8 databases to identify randomized controlled trials (RCTs) evaluating acupuncture interventions for FMS. We used ROB 2.0 tool to assess the risk of bias in selected studies. Heterogeneity among the studies was detected using the *I*^2^ test. Identifying sources of heterogeneity using subgroup analysis. Sensitivity analysis was performed to assess the stability of the results. Meta-analysis was performed using Stata 15.1.

**Results:**

17 RCTs involving 1,066 patients were included. Meta-analysis results showed that the intervention group had significantly lower VAS scores (SMD: −0.77; 95% CI: −1.00, −0.55), FIQ scores (SMD: −0.98; 95% CI: −1.43, −0.53), and the number of pain points (SMD: −1.36; 95% CI: −1.65, −1.08). It also improved depression and fatigue (SMD: −0.78; 95% CI: −1.10, −0.47) and fatigue (SMD: −0.51; 95% CI: −0.72, −0.30), *P* < 0.05, but did not significantly improve sleep quality (*P* > 0.05).

**Conclusions:**

Acupuncture can reduce pain, improve depression and fatigue, and overall lower FIQ scores in the treatment of FMS. These findings require further confirmation through larger-scale, high-quality studies.

**Systematic review registration:**

Identifier CRD20251120515

## 1 Introduction

FMS is a syndrome characterized by chronic generalized skeletal muscle pain (1). According to the revised diagnostic criteria of the American College of Rheumatology (ACR) in 2016, the pain associated with FMS must meet systemic criteria: persistent pain in at least four of the five regions (limbs and axial region), with the pain often being diffuse in nature, and symptoms persisting for more than 3 months (2). In addition to pain, FMS is associated with a cluster of characteristic symptoms, such as fatigue, sleep disturbances, and anxiety or depression (3, 4). FMS is the third most common musculoskeletal disorder in terms of prevalence, following low back pain and osteoarthritis. Its prevalence increases with age, peaking among individuals aged 50–60. Individuals with FMS require nearly twice as many annual medical visits as healthy individuals, and their overall healthcare costs are estimated to be three times higher than those of the general population (5).

Currently, pharmacological treatment for FMS primarily targets symptom control, such as pain, using conventional analgesics like pregabalin, duloxetine, tramadol, and amitriptyline. However, pharmacological treatment has limitations, with only 30–50% of patients responding to medication, and pain relief often less than 50% (6); Etoricoxib did not show superiority over placebo in randomized controlled trials. Additionally, there are significant drug side effects, such as dizziness and weight gain from pregabalin, and nausea, insomnia, and sexual dysfunction from duloxetine (7). Given the limitations of drug efficacy and side effects, there is a need to explore non-pharmacological therapies to reduce drug usage and potentially replace drug therapy to some extent.

Acupuncture, as one of the non-pharmacological therapies for FMS, is recommended for treating FMS patients with pain as the primary symptom (8). Studies suggest that acupuncture may improve central sensitization by increasing serum neuropeptide Y levels, thereby reducing pain in fibromyalgia (9). Electroacupuncture can downregulate inflammatory factors such as interleukin-1, TNF-α, interferon-γ in mouse plasma, as well as inhibit the vanilloid subtype 1 channel of the transient receptor potential family, thereby alleviating pain in FMS patients (10, 11). Currently, there is still a lack of multicenter, large sample RCTs, and there is not enough evidence to demonstrate that acupuncture improves pain and associated symptoms in FMS patients.

Therefore, this study employs systematic reviews and meta-analysis to verify whether acupuncture can effectively alleviate symptoms such as pain, fatigue, and depression in patients with fibromyalgia syndrome, thereby providing scientific evidence for clinical application.

## 2 Methods

This study was a systematic review and meta-analysis of the literature, and have been registered in the PROSPERO Registry (CRD420251120515). This study was conducted in accordance with Cochrane recommendations and follows the PRISMA guidelines (12).

### 2.1 Inclusion and exclusion criteria

#### 2.1.1 Study type

RCTs.

#### 2.1.2 Study population

Patients diagnosed with FMS, regardless of disease duration, occupation, age, nationality, etc.

#### 2.1.3 Intervention measures

The intervention group used acupuncture and the control group used conventional pain medications (tramadol hydrochloride sustained-release tablets, amitriptyline, pregabalin, and ibuprofen), sham acupuncture, or no intervention.

#### 2.1.4 Outcome measures

Primary outcomes: (1) VAS (13–25): patients marked their subjective pain level on a 10-centimeter linear scale, and physicians measured the marked point to obtain the pain score; (2) Fibromyalgia Impact Questionnaire (FIQ) (13–15, 17–19, 23, 26–28): a self-assessment questionnaire designed to evaluate the extent to which FMS impacts an individual's daily life. The questionnaire consists of ten questions, with a maximum score of 100 points. A higher score indicates a more significant impact; (3) Number of pain points (13, 22, 24, 25, 27): number of pain points at pressure below 4 kg/cm^2^; Secondary outcomes: (4) Depression (13–16, 18, 20, 21, 25, 27): different depression scales were used to assess the severity of depression in FMS patients, with higher scores indicating more severe depression; (5) Sleep (16, 20, 21, 23, 27, 28): sleep quality in FMS patients were assessed using various sleep scoring scales, with higher scores indicating more severe insomnia; (6) Fatigue (14–16, 18, 27–29): fatigue levels in FMS patients were assessed using various fatigue rating scales, with higher scores indicating more pronounced fatigue.

#### 2.1.5 Exclusion criteria

(1) Studies not related to FMS; (2) Reviews and conferences; (3) Duplicate studies or studies with identical data; (4) Studies that cannot be analyzed without original data; (5) Studies not in Chinese or English.

#### 2.1.6 Research question

Is acupuncture more effective than conventional pain medications, sham acupuncture, or no intervention for pain, overall function, depression, fatigue, and sleep in patients with FMS?

### 2.2 Literature search

Computerized databases including Web of Science, PubMed, The Cochrane Library, Embase, CNKI, CBM, VIP, and WanFang were searched using a combination of subject terms and free-text keywords, with adjustments made according to the characteristics of each database. Search terms included: “fibromyalgia,” “fibromyalgia syndrome,” “acupuncture,” and “acupuncture therapy,” among others. The search time range spans from the establishment date of each database to July 2025.

### 2.3 Literature screening and data extraction

Two researchers independently screened the literature, extracted the data and cross-checked them. If there is a disagreement, it shall be settled through discussion or consultation with a third party. During literature screening, duplicate documents were first excluded. Then, the titles were read, and documents clearly unrelated to the study were excluded. Subsequently, the abstracts were read, and finally, the full texts were reviewed to determine inclusion. Data extraction included: (1) study authors and publication year; (2) sample size; (3) age; (4) treatment duration; (5) intervention measures; (6) outcome measures.

When raw data is unavailable or data is presented in graphical form, attempts will be made to contact the relevant authors to obtain the raw data, and GetData Graph Digitizer 2.26 will be used to extract data from the graphs.

### 2.4 Risk of bias

The Cochrane ROB tool 2.0 (30) was used to assess the risk of bias. Evaluation results were categorized as “low risk,” “unclear,” or “high risk.” The methodological quality was evaluated independently by two evaluators. If there was a disagreement, the agreement was reached according to the third party's opinion.

### 2.5 Quality assessment

Used GRADEpro GDT web application (https://www.gradepro.org) (31) to assess the quality of evidence for outcomes. The quality of evidence for outcomes was assessed based on the GRADE guidelines (gradeworkinggroup.org). The overall evidence for each outcome measure. GRADE downgrades evidence quality based on five factors: risk of bias, inconsistency, indirectness, imprecision, and other considerations. Risk of bias: included studies exhibit methodological limitations (e.g., inadequate randomization, allocation concealment, or blinding). Inconsistency: results across studies show substantial variation without plausible explanation. Imprecision: the 95% confidence interval for the effect size is excessively wide, or the total sample size is too small. Indirectness: evidence is not directly relevant to the clinical question in terms of PICO. Other considerations: overestimation of pooled results due to non-publication of studies with negative or ineffective outcomes. This process is conducted independently by two assessors, with consensus reached through discussion or third-party consultation.

### 2.6 Statistical analysis

State 15.1 was used for meta-analysis. All data in this study were continuous variables. Due to inconsistencies in units and observation times among the indicators, different scales are used for some indicators (depression, sleep, fatigue), standardized mean differences (SMD) were used to represent the results, with confidence intervals (CI) set at 95%. Heterogeneity was detected by *I*^2^ test results. The *I*^2^ statistic quantifies the proportion of total variation attributable to between-study variation rather than sampling error. Its formula is: *I*^2^ = max (0%, [(*Q* – *df* )/*Q*] × 100%), where *Q* is the heterogeneity chi-square statistic and df is the degrees of freedom. When *I*^2^ ≤ 50%, a fixed-effects model (FEM) based on the inverse variance method was employed, when *I*^2^ > 50%, a random-effects model (REM) based on the DerSimonian– (58). Subgroup meta-analysis was used to find the source of heterogeneity, and sensitivity analysis was performed to evaluate the stability of the meta-analysis results. To quantitatively explore potential sources of study heterogeneity, we conducted meta-regression analysis for indicators exhibiting high heterogeneity with more than 10 included studies. Additionally, when the number of studies on an outcome exceeds 10, the funnel plot and Egger's analysis were used to detect publication bias of that outcome measure.

## 3 Results

### 3.1 Search result

**Figure 1:**
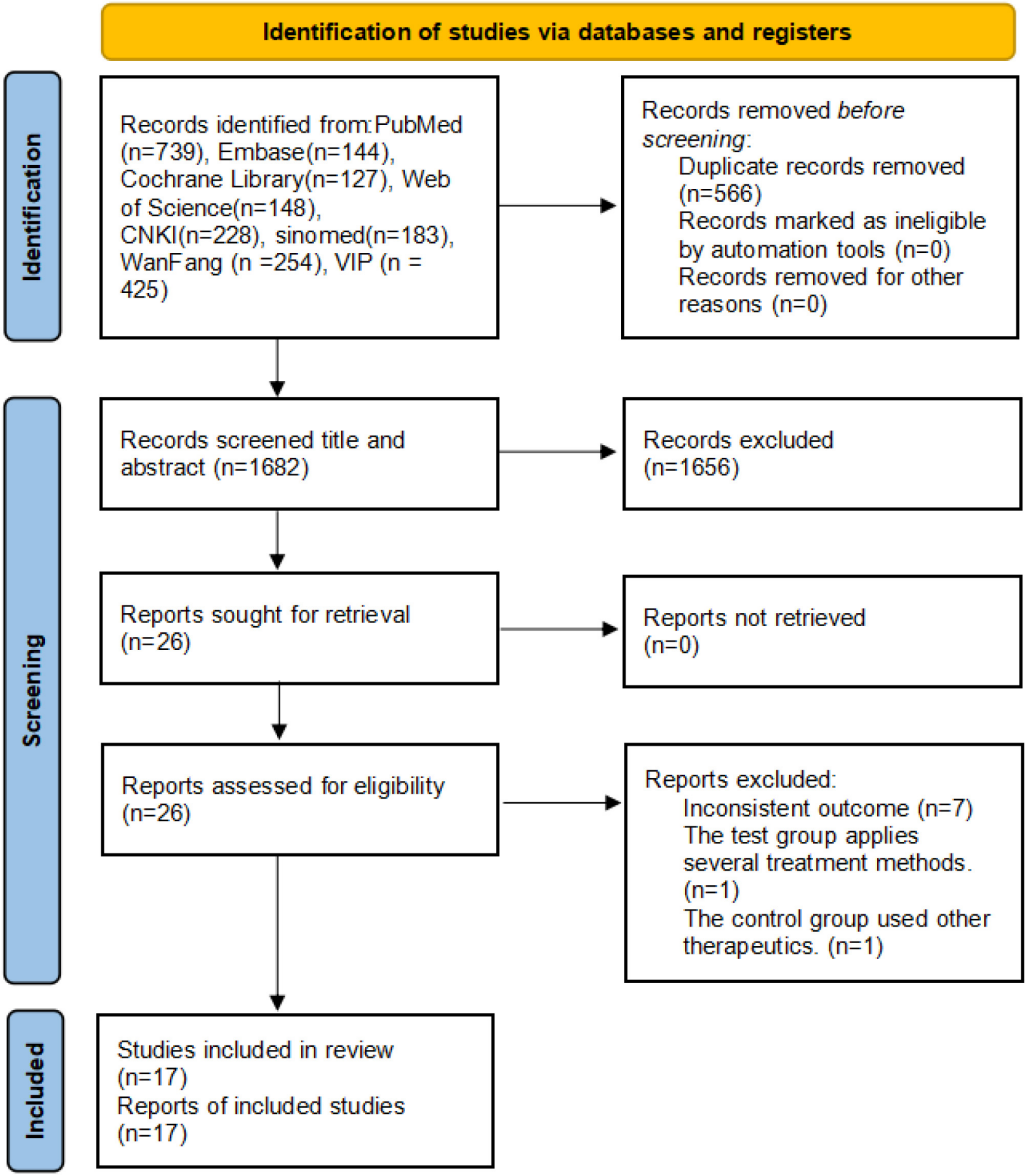
PRISMA statement flow chart.

We preliminarily retrieved 2,248 studies from databases. We summarized the retrieved studies, excluded 566 duplicate studies, and first screened them by reading the titles and abstract, then removed non-FMS, non-RCTs, non-acupuncture studies, and reviews. Next, by reading the full text, we excluded 1 study where the experimental group used other acupuncture methods; seven studies lacking useful data; and 1 study where the control group used other intervention measures. Finally, this study included 17 studies (13–29) for a meta-analysis (Figure 1). The intervention group received acupuncture, while the control group received conventional pain medication, sham acupuncture, or no intervention. The treatment course is 2–12 weeks, mainly concentrated at 4 weeks (Table 1).

**Table 1:** Basic information of the studies.

**Study**	**Sample size (T/C)**	**Age (y), Mean ±SD**	**Interventions**		**Frequency**	**Outcomes**
			**T**	**C**		
(13)	24/25	34.71 ± 6.09/34.20 ± 6.84	Acupuncture	Sham acupuncture	Twice a week, 8 times in total	①②③④
(14)	25/24	/	Acupuncture	Sham acupuncture	20 min, twice a week, 6 times in total	①②④⑥
(15)	25/25	47.28 ± 7.86/43.60 ± 8.18	Acupuncture	Sham acupuncture	30 min, 3 treatments in the first week, twice a week for 2 weeks, then once a week for 5 weeks, 12 times in total	⑥
(29)	22/15	46 ± 10.1/48.1 ± 10.9	Acupuncture	Sham acupuncture	20 min, once a week for 3 weeks, twice a week for 3 weeks, three times a week for 3 weeks, for a total of 18 times.	①④⑤⑥
(16)	25/25	/	Acupuncture	Sham acupuncture	Twice a week, 24 times in total	①②
(17)	80/82	52.3 ± 9.6/53.2 ± 9.6	Acupuncture	Sham acupuncture	Once a week for 9 weeks	①②④⑥
(18)	34/33	56.15 ± 7.9/54.39 ± 8.2	Acupuncture	No intervention	30 min, twice a week for 5 weeks	①②
(19)	26/25	45.5 ± 7.5/44.2 ± 6.8	Acupuncture	Sham acupuncture	Twice a week for 5 weeks	②
(18)	34/33	56.15 ± 7.9/54.39 ± 8.2	Acupuncture	No intervention	30 min, twice a week for 5 weeks	②③④⑤⑥
(27)	15/15	43.86 ± 7.9/44.2 ± 10.8	Acupuncture	Fluoxetine	30 min, three times a week for 2 weeks	②⑤⑥
(28)	40/43	42.35 ± 6.86/45.52 ± 7.43	Acupuncture	Tramadol+ Amitriptyline	20 min, three times a week for 4 weeks	①④⑤
(20)	30/30	35 ± 8/34 ± 6	Acupuncture	Amitriptyline	30 min, once a day for 2 weeks; three times a week for 2 weeks; twice a week for 8 weeks	①③
(21)	41/41	42 ± 10/43 ± 10	Acupuncture	Prelamine	30 min, once a day for 4 weeks	①②⑤
(22)	19/19	50 ± 2.9/49 ± 3.4	Acupuncture	Amitriptyline	30 min, once a day for 4 weeks	①③
(23)	42/39	/	Acupuncture	Amitriptyline	30 min, three times a week for 12 weeks	①③④
(24)	30/30	/	Acupuncture	Ibuprofen	6 min, once a day for 2 weeks	①③
(25)	25/25	43.3 ± 3.6/42.3 ± 4.2	Acupuncture	Amitriptyline	30 min, three times a week for 6 weeks	①③④

VAS; FIQ; Number of pain points; Depression; Sleep; Fatigue.

### 3.2 Risk of bias

**Figure 2:**
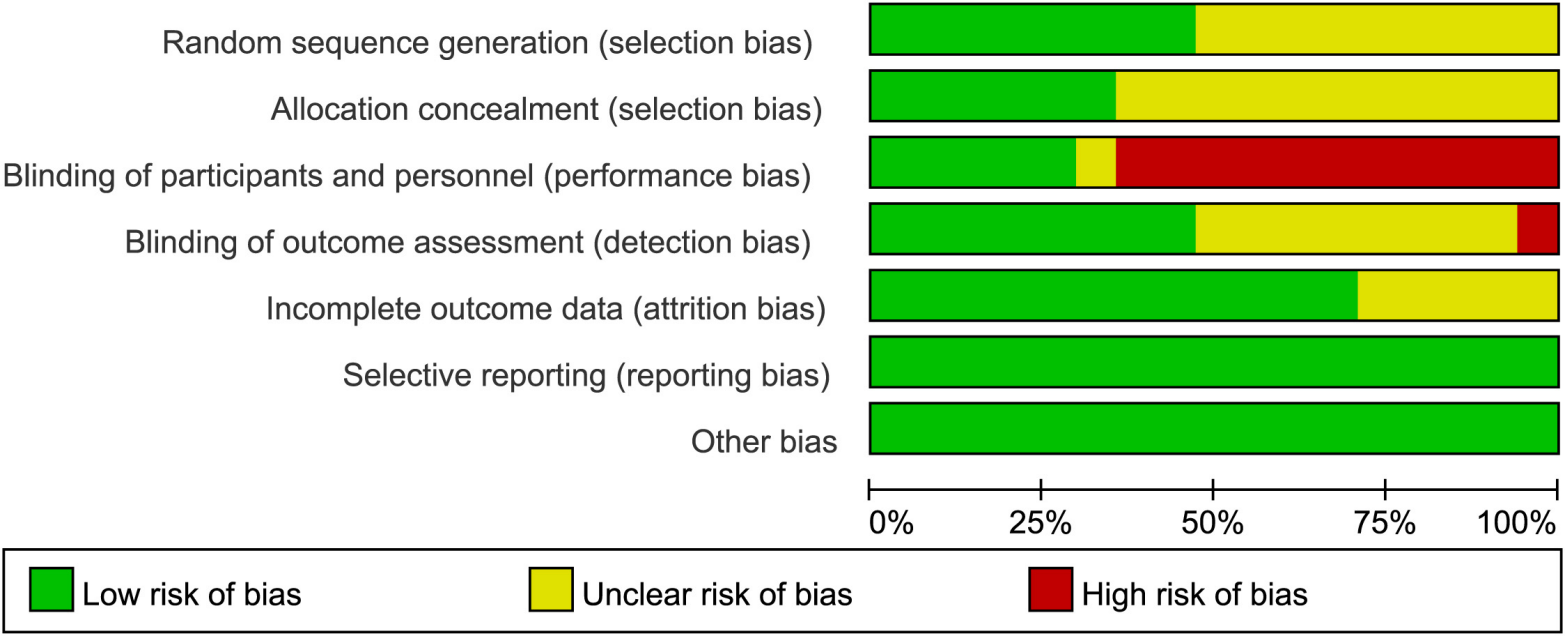
Risk of bias.

The random number table method was properly implemented in 8 studies (15–19, 26, 27, 29), the method of randomization was not reported in the remaining ones. Six studies (15–18, 26, 27, 29) employed appropriate allocation concealment methods; whereas, the methodology for concealment was omitted in the remaining studies. Five studies (13, 14, 16, 17, 29) implemented blinding for both patients and operators, 1 study (19) did not mention blinding, and the remaining studies did not implement blinding. In the evaluation of outcome measures, 8 studies (13, 14, 16–18, 26, 27, 29) used appropriate blinding methods, 1 study (15) mentioned that blinding was not used, the remaining studies did not state whether blinding was implemented. All studies were judged to be at low risk of bias for selective reporting, as they provided complete outcome reporting (Figure 2 and Supplementary Figure S1).

### 3.3 Meta-analysis

#### 3.3.1 VAS

**Figure 3:**
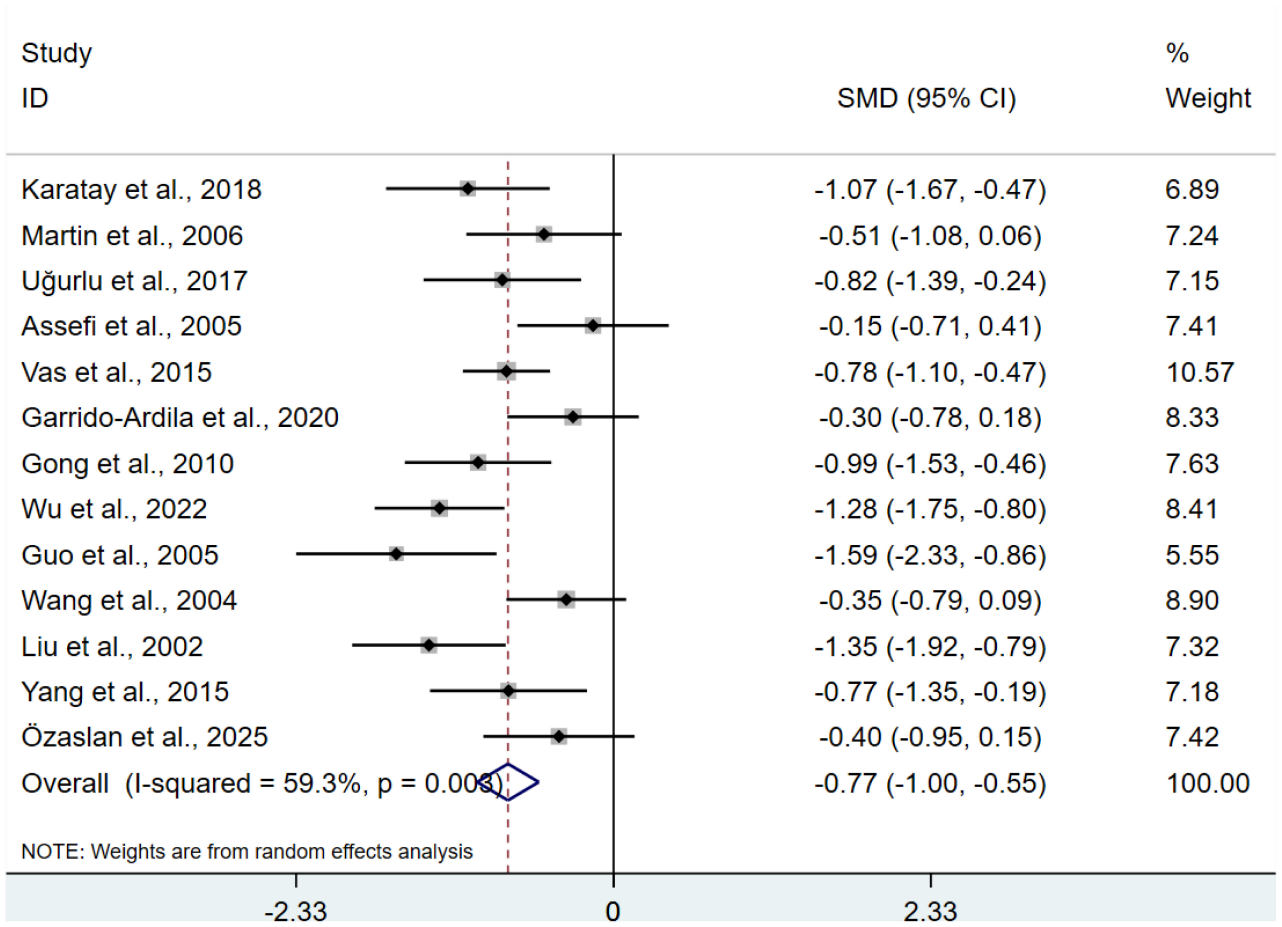
Meta-analysis of VAS.

A total of 13 studies (13, 15–25) involving 849 patients were included. Heterogeneity testing revealed *I*^2^ = 59.3%, indicating moderate heterogeneity, and a REM was used for analysis. Meta-analysis showed that after acupuncture treatment, the VAS in the intervention group were significantly lower than those in the control group (SMD: −0.77; 95% CI: −1.00, −0.55), τ^2^ = 0.0994, with statistical significance (*P* < 0.05; Figure 3).

#### 3.3.2 FIQ

**Figure 4:**
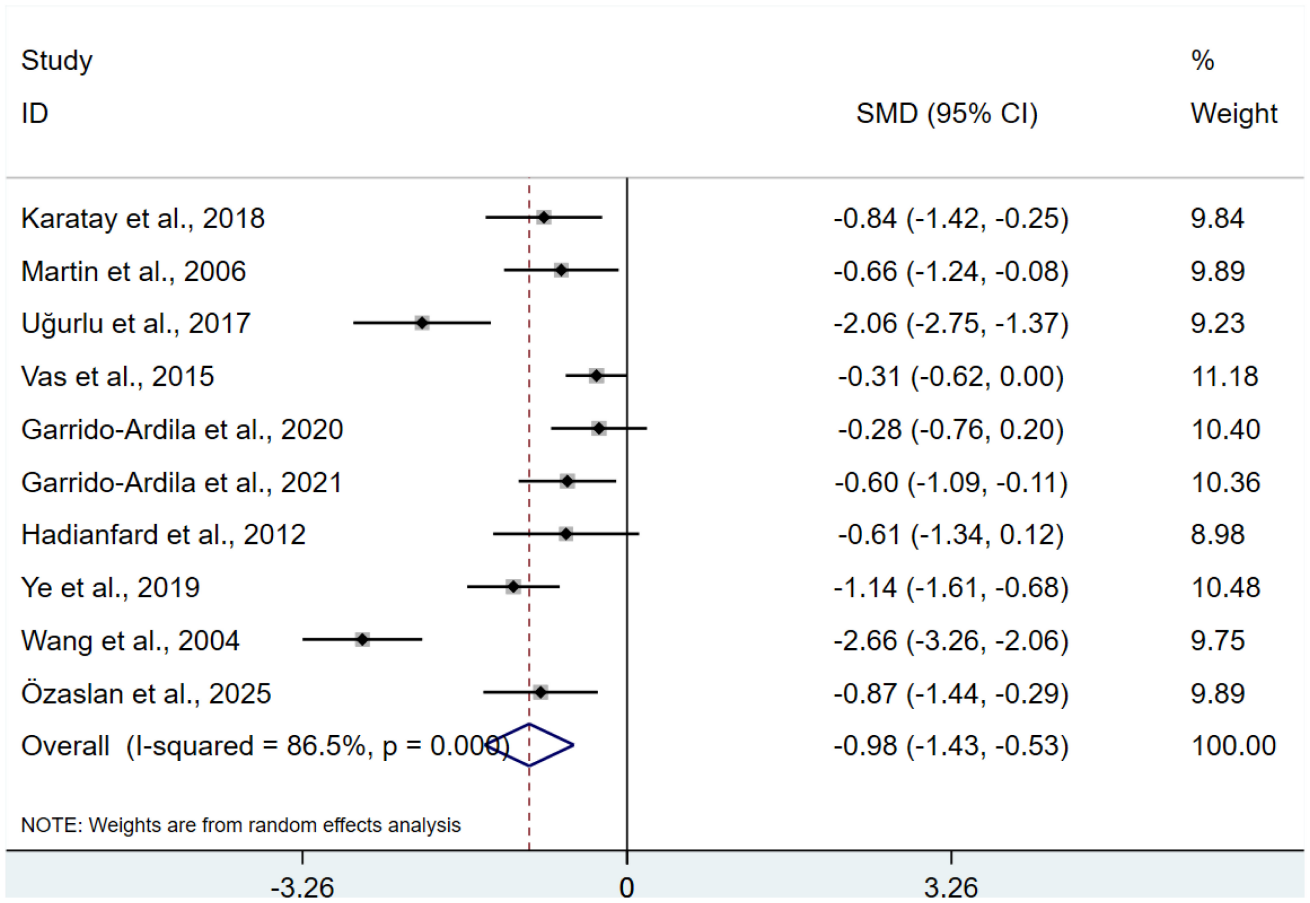
Meta-analysis of FIQ.

A total of 10 studies (13–15, 17–19, 23, 26–28), involving 689 patients. Heterogeneity testing revealed *I*^2^ = 86.5%, indicating the presence of heterogeneity, and a REM was used for analysis. Results of the meta-analysis indicated that FMS patients receiving acupuncture exhibited significantly lower FIQ than control group (SMD: −0.98; 95% CI: −1.43, −0.53), τ^2^ = 0.4444, with statistical significance (*P* < 0.05; Figure 4).

#### 3.3.3 Number of pain points

**Figure 5:**
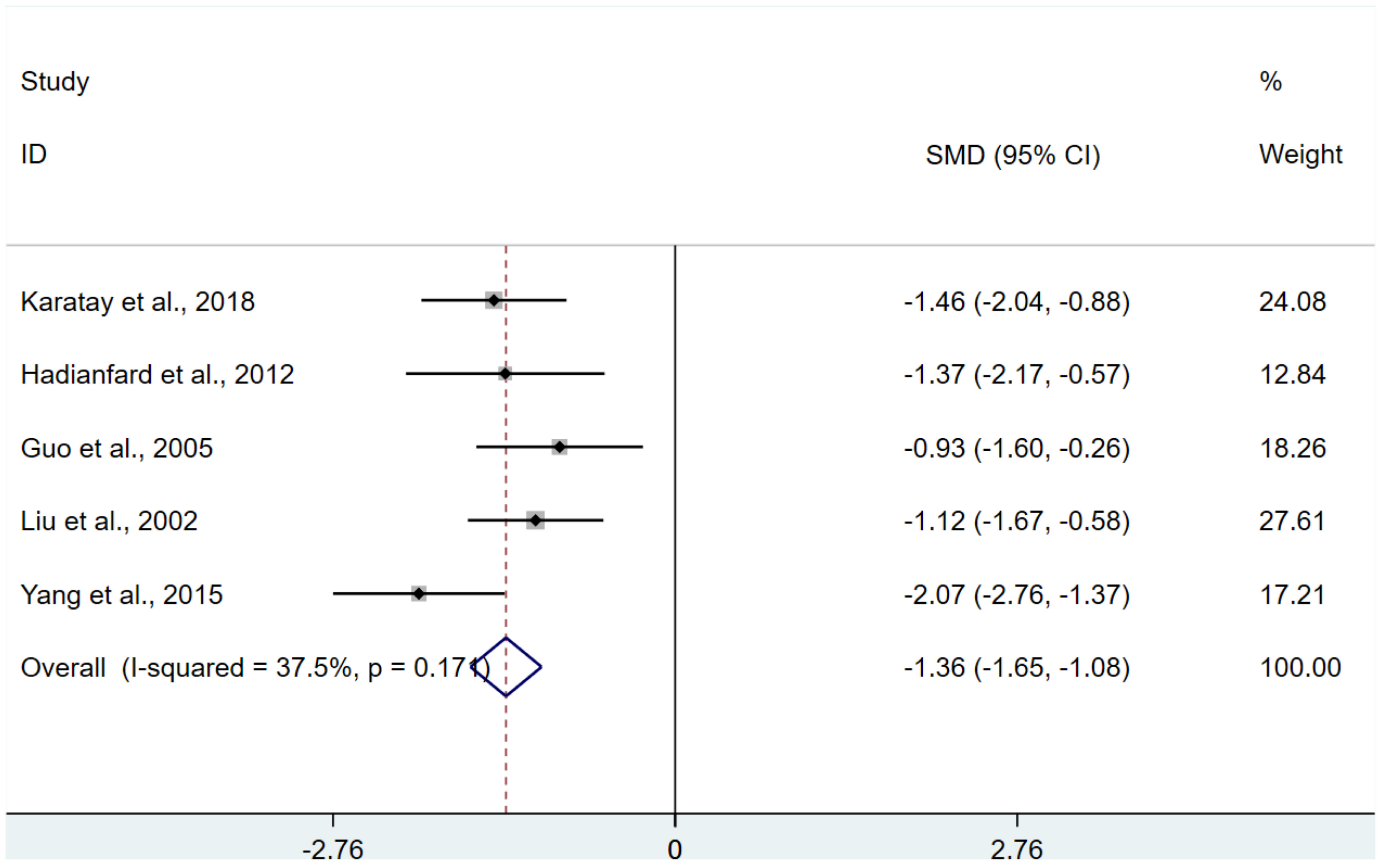
Meta-analysis of number of pain points.

A total of 5 studies (13, 22, 24, 25, 27) were included, involving 237 patients regarding the number of pain points. Heterogeneity testing showed I^2^ = 37.5%, and a FEM was used for analysis. Results of the meta-analysis indicated a significant decrease in pain points among FMS patients receiving acupuncture relative to control group (SMD: −1.36; 95% CI: −1.65, −1.08), with a statistically significant difference (P < 0.05; Figure 5).

#### 3.3.4 Depression

**Figure 6:**
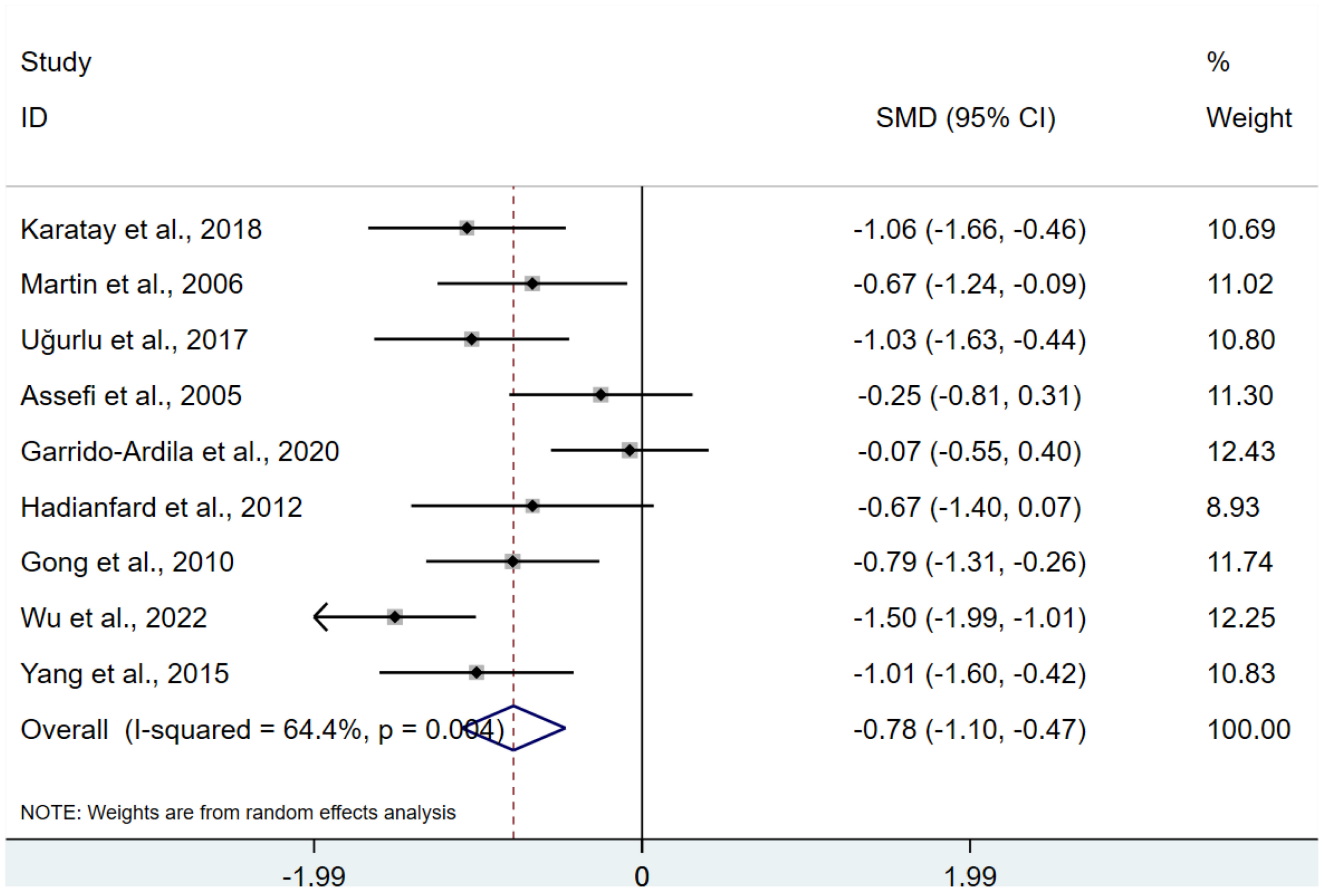
Meta-analysis of depressions.

A total of 9 studies (13–16, 18, 20, 21, 25, 27) were included, involving 487 patients with depression. Heterogeneity testing revealed I^2^ = 64.4%, indicating moderate heterogeneity. Analyses were performed using a REM. The results indicated that the intervention group achieved a superior improvement in depression relative to the control group after acupuncture intervention (SMD: −0.78; 95%Cl: −1.10, −0.47), τ^2^ = 0.1484, with a statistically significant difference (P < 0.05; Figure 6).

#### 3.3.5 Sleep

**Figure 7:**
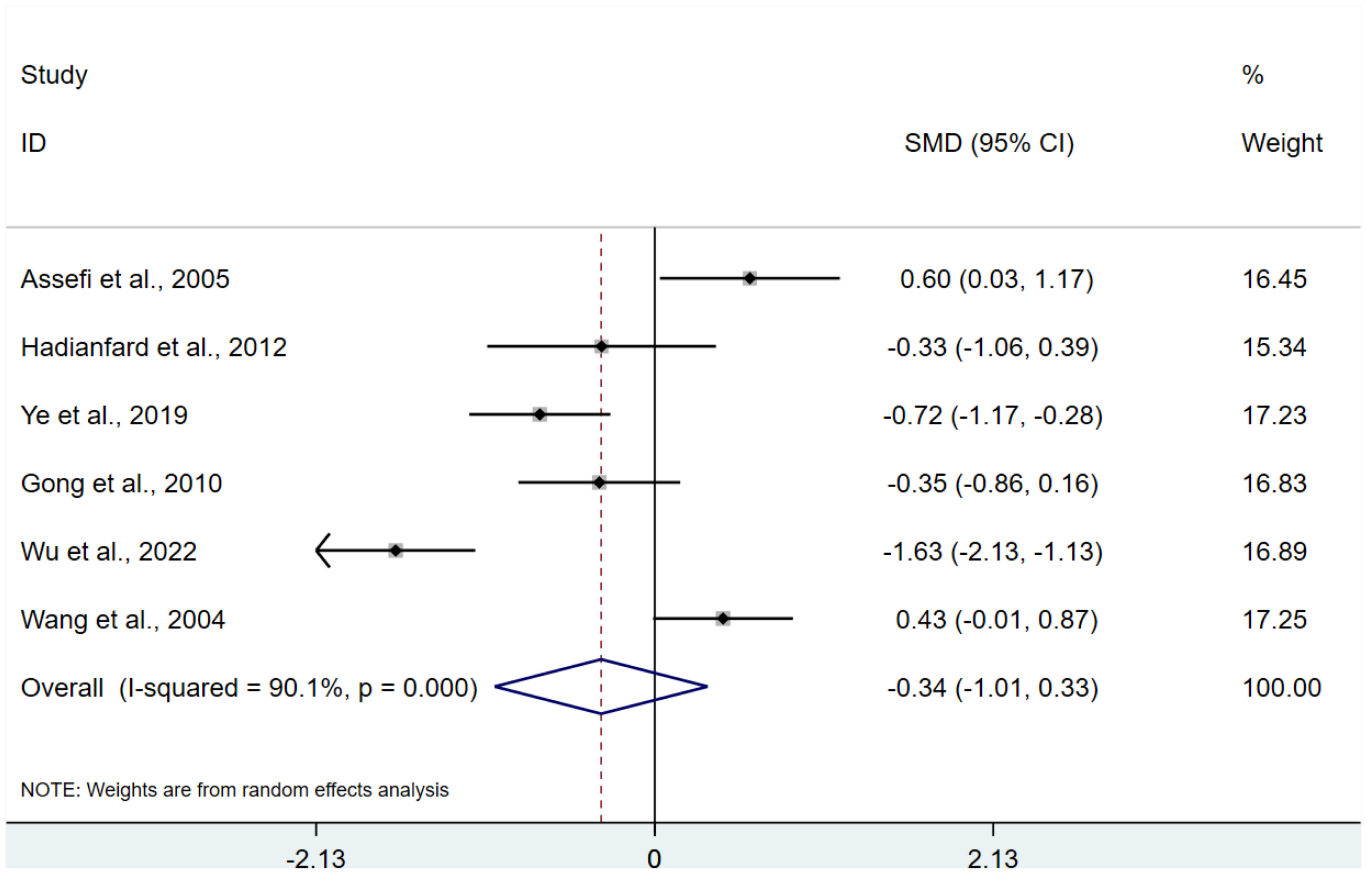
Meta-analysis of sleep.

There were 6 studies (16, 20, 21, 23, 27, 28) involving 386 patients related to sleep. Heterogeneity testing showed *I*^2^ = 90.1%, indicating heterogeneity, so a REM was used for analysis. Meta-analysis showed that after acupuncture treatment, there was no statistically significant difference in sleep between the intervention group and the control group (SMD: −0.34; 95% CI: −1.01, 0.33), τ^2^ = 0.6293 (*P* > 0.05; Figure 7).

#### 3.3.6 Fatigue

**Figure 8:**
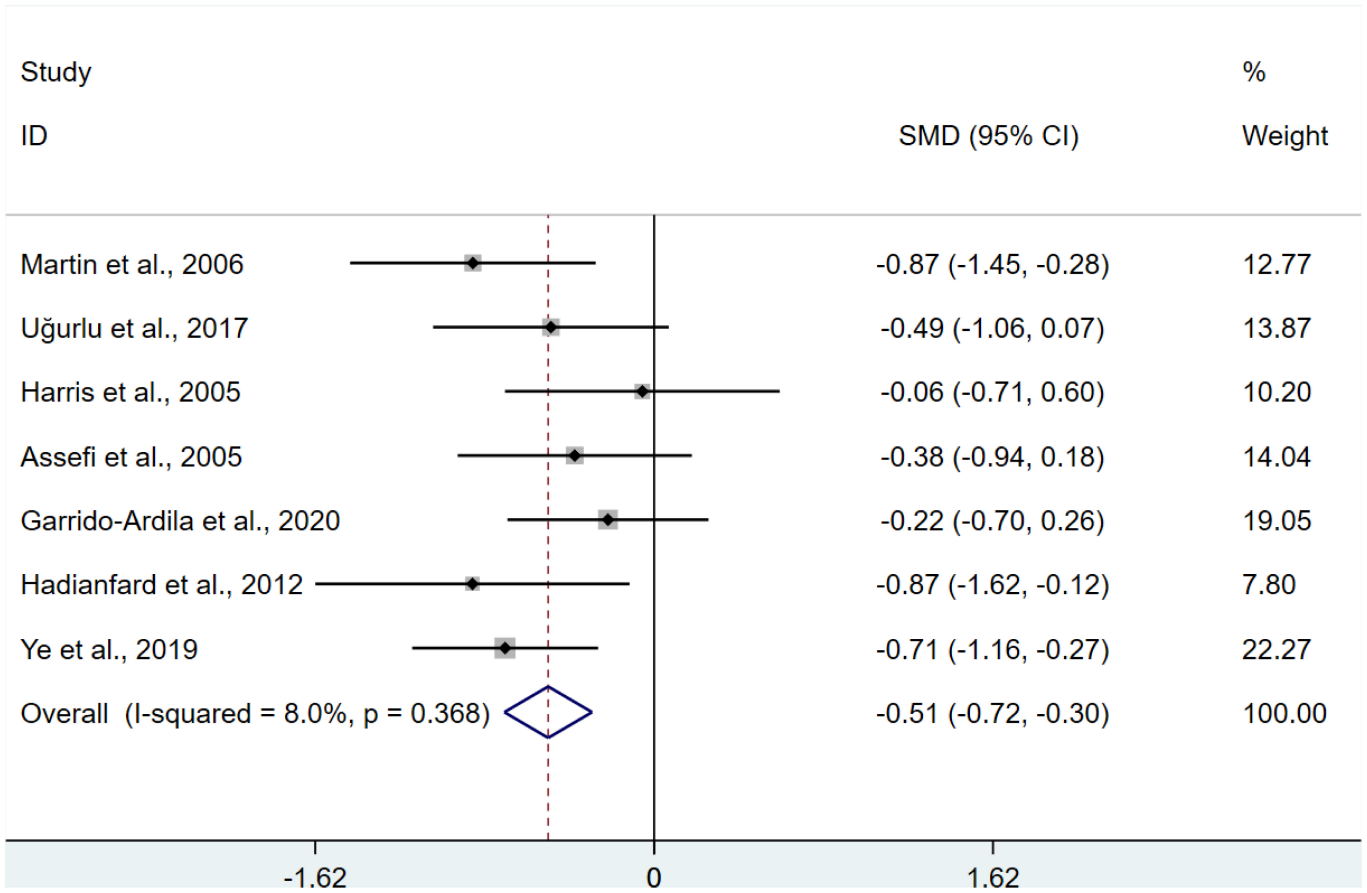
Meta-analysis of fatigue.

A total of 7 studies (14–16, 18, 27–29) were identified, involving 366 patients with fatigue. Heterogeneity testing revealed *I*^2^ = 8.0%, indicating low heterogeneity, and a FEM was used for analysis. Meta-analysis revealed a significantly greater improvement in fatigue in the intervention group compared to the control group following acupuncture treatment (SMD: −0.34; 95%Cl: −1.01, 0.33), (*P* < 0.05; Figure 8).

### 3.4 Subgroup meta-analysis

Significant heterogeneity was detected across the outcomes (VAS, FIQ, depression, and sleep). To investigate the sources of this heterogeneity, subgroup analysis were explored by treatment course and different intervention measures in the control group.

#### 3.4.1 VAS

Subgroup analysis by intervention duration demonstrated that, at 2–4 weeks, 5–6 weeks, and 8–12 weeks, acupuncture exhibited substantially lower VAS scores compared to the control group (SMD: −1.11; 95% CI: −1.34, −0.88; *I*^2^ = 31.6%), (SMD: −0.46; 95% CI: −0.77, −0.16; *I*^2^ = 0.0%), and (SMD: −0.58; 95% CI: −0.80, −0.37; *I*^2^ = 46.5%), with all differences reaching statistical significance (*P* < 0.05). The reduction of heterogeneity suggested that different treatment durations in the intervention group may be the source of heterogeneity (Supplementary Figure S2).

#### 3.4.2 FIQ

A subgroup meta-analysis of the FIQ acupuncture intervention course revealed that, at 2–4 weeks, 5–6 weeks, and 8–12 weeks, acupuncture showed superior outcomes in FIQ compared to control group (SMD: −0.87; 95% CI: −1.16, −0.59; *I*^2^ = 0.0%), (SMD: −0.56; 95% CI: −0.88, −0.23; *I*^2^ = 17.1%), and (SMD: −1.66; 95% CI: −3.27, −0.04; *I*^2^ = 96.5%), demonstrating a marked improvement that reached statistical significance (*P* < 0.05). According to the subgroup analysis, the heterogeneity of 2–4 weeks and 5–6 weeks decreased significantly compared with the previous one, indicating that the course of 8–12 weeks may be the source of heterogeneity (Supplementary Figure S3).

#### 3.4.3 Depression

A subgroup analysis was conducted on different intervention measures (sham acupuncture, medication, no intervention) in the control group. The results showed that acupuncture significantly improved depression compared to sham acupuncture and medication (SMD: −0.74; 95% CI: −1.12, −0.36; *I*^2^ = 41.3%), (SMD: −1.03; 95% CI: −1.41, −0.65; *I*^2^ = 43.1%), the differences were statistically significant (*P* < 0.05). Since only one study had a no intervention control group, it was not meaningful for comparison. After subgroup analysis, heterogeneity decreased compared to before, suggesting that different intervention measures in the control group may be a source of heterogeneity (Supplementary Figure S4).

#### 3.4.4 Sleep

A subgroup analysis of the acupuncture for sleep showed that at 2–4 weeks and 8–12 weeks, the intervention group had significantly better sleep outcomes compared to the control group (SMD: −0.78; 95% CI: −1.38, −0.18; *I*^2^ = 80.4%), (SMD: 0.49; 95% CI: 0.15, 0.84; *I*^2^ = 0.0%), the differences were statistically significant (*P* < 0.05). Subgroup analysis showed that heterogeneity decreased significantly between 8–12 weeks compared to 2–4 weeks, suggesting that the 2–4 week treatment course may be the source of heterogeneity (Supplementary Figure S5).

### 3.5 Meta-regression analysis

To investigate the sources of high heterogeneity in VAS, we performed meta-regression analysis. For VAS, we incorporated treatment duration into the model. Meta-regression results showed *P* = 0.009 < 0.05, indicating that treatment duration significantly influenced outcomes and was thus a source of heterogeneity (Supplementary Figure S6).

### 3.6 Sensitivity analysis

We performed a sensitivity analysis to assess the robustness of the findings across the six key metrics (VAS, FIQ, number of pain points, depression, sleep, and fatigue). The exclusion of any single study from the analysis had no substantial impact on the magnitude of the pooled effect estimate, the SMD value differed little from the original overall SMD value, and statistical significance was maintained with the *P*-value consistently remaining below 0.05, indicating that the meta-analysis results of the above six indicators are robust and reliable (Supplementary Figure S7).

### 3.7 Publication bias

The inverted funnel plot for VAS and FIQ showed that all studies are within the 95% CI and are symmetrically distributed, suggesting a low possibility of publication bias for VAS and FIQ (Egger analysis: *P* = 0.575, 0.083 > 0.05; Supplementary Figure S8).

### 3.8 Certainty assessment

The assessment of evidence quality was conducted using the GRADE approach (Table 2). The results showed that the evidence grade for VAS, FIQ, depression, and sleep was rated as low due to the inclusion of studies with heterogeneity and a risk of bias in the results. However, there were no issues with other aspects, so their evidence grades were rated as moderate. The number of pain points included studies at risk of bias, with a sample size of no more than 300, so the evidence grade was rated as low. Fatigue included articles at risk of bias, but there were no other issues, so the evidence grade was rated as moderate.

**Table 2:** Certainty assessment.

**Outcomes**	**Number of studies**	**Design**	**Risk of bias**	**Inconsistency**	**Indirectness**	**Imprecision**	**Other considerations**	**Number of patients**		**Effect**	**Quality**
								**Intervention group**	**Control group**	**SMD (95%CI)**	
VAS	13	Randomized trials	Serious	Serious	No serious indirectness	No serious imprecision	No serious	426	423	−0.75 (−0.99, −0.50)	Low
FIQ	10	Randomized trials	Serious	Serious	No serious indirectness	No serious imprecision	No serious	345	344	−0.98 (−1.43, −0.53)	Low
Number of pain points	5	Randomized trials	Serious	No serious inconsistency	No serious indirectness	Serious	No serious	113	124	−1.36 (−1.65, −1.08)	Low
Depression	9	Randomized trials	Serious	Serious	No serious indirectness	No serious imprecision	No serious	244	243	−0.78 (−1.10, −0.47)	Low
Sleep	6	Randomized trials	Serious	Serious	No serious indirectness	No serious imprecision	No serious	193	193	−0.34 (−1.01, 0.33)	Low
Fatigue	7	Randomized trials	Serious	No serious inconsistency	No serious indirectness	No serious imprecision	No serious	186	180	−0.51 (−0.72, −0.30)	Moderate

## 4 Discussion

This analysis encompassed 17 RCTs with a total of 1,066 participants to assess the efficacy of acupuncture for FMS. The meta-analysis results showed that the treatment group had a significantly greater reduction in VAS (SMD: −0.77; 95% CI: −1.00, −0.55), FIQ (SMD: −0.98; 95% CI: −1.43, −0.53), the number of pain points (SMD: −1.36; 95% CI: −1.65, −1.08), and improved depression and fatigue (SMD: −0.78; 95% CI: −1.10, −0.47), (SMD: −0.51; 95% CI: −0.72, −0.30), *P* < 0.05, but did not significantly improve sleep quality (*P* > 0.05).

Compared with recent similar studies, both this research and Valera-Calero's review (32) employed control group settings closer to clinical practice, such as conventional analgesic medications or no intervention. This enhanced the clinical applicability of their conclusions in real healthcare decision-making environments. However, this design also introduces higher heterogeneity. In contrast, Zheng 's study (33) strictly limited the control group to sham and simulated or placebo acupuncture to validate acupuncture's specific therapeutic effects. While this approach yields higher internal validity, its conclusions may be conservative due to potent placebo effects, resulting in relatively limited external validity. Second, findings on sleep improvement vary across studies. This research found no significant benefit for sleep enhancement, whereas Valera-Calero's analysis indicated short-term sleep improvements with dry needling and acupuncture. Such inconsistencies may stem from differences in treatment duration, follow-up periods, assessment tools, and acupoint prescriptions.

FMS is a chronic condition primarily characterized by widespread pain. This complex syndrome frequently presents with a constellation of co-occurring symptoms, including depression, sleep disorders, and fatigue (3). Symptoms are caused by the interaction of multiple factors, including central sensitization, neurotransmitter imbalance, neuroinflammation, HPA axis dysfunction, and mitochondrial metabolic abnormalities (34). Acupuncture provides a safe and effective treatment option for FMS through its multi-targeted, multi-level integrated regulatory effects.

Pain is a prominent manifestation of FMS, and the primary pathological mechanism underlying FMS pain is central sensitization, characterized by disrupted processing and perception of pain signals in the central nervous system (35). This process was driven by neurotransmitter imbalance, glial cell activation, inflammatory factor dysregulation, and peripheral-central signal interaction (36–38). The key pathological feature of central sensitization is an imbalance between excitatory and inhibitory neurotransmitters. In animal studies of FMS, excitatory neurotransmitters such as substance *P* and glutamate were significantly elevated in the dorsal horn of the spinal cord, while inhibitory neurotransmitters like serotonin and gamma-aminobutyric acid (GABA) are reduced (39). In clinical studies, a fMRI studies have shown that neuronal activation in these regions is significantly higher than in healthy individuals, and increased connectivity in the periventricular gray matter leads to a lowered pain threshold and abnormal sensitivity to mild stimuli (40). A significant dysregulation exists between excitatory and inhibitory neurotransmission within the central nervous system of individuals with FMS. Studies demonstrate that cerebrospinal fluid levels of substance *P* are markedly elevated (2–3 fold) in FMS patients compared to healthy controls, GABA levels in the insular cortex are reduced by approximately 30%, and glutamate levels are significantly elevated (41). A study found (42) that acupuncture treatment for chronic pain patients specifically enhances functional connectivity between the primary somatosensory cortex and the anterior insula. Acupuncture promotes the activation of the endogenous analgesic system to reduce pain. Acupuncture stimulation activates Aδ and C-type afferent nerve fibers, transmitting signals to the dorsal horn of the spinal cord, thereby activating brain regions such as the periaqueductal gray matter of the midbrain and the ventromedial nucleus of the medulla. The activation of these regions enhances the descending inhibitory pathways, releasing inhibitory neurotransmitters, thereby inhibiting the transmission of nociceptive signals in the dorsal horn of the spinal cord (43, 44). Glial cell activation perpetuates central sensitization in FMS. In FMS model rats, the number of GFAP-positive cells in the dorsal horn of the spinal cord increased by more than 50% compared to normal rats. Activated astrocytes further disrupt inflammatory factors, exacerbating central sensitization (39). Additionally, dysfunction of the HPA axis in FMS patients leads to disrupted cortisol release rhythms, elevated pro-inflammatory cytokines, and further promotes central sensitization (3, 45). Huang Yuting's research revealed that following electroacupuncture intervention, the number of GFAP-positive cells significantly decreased (*P* < 0.05), while simultaneously reducing spinal cord TNF-α levels. This suggests that suppressing astrocyte activation can diminish the release of inflammatory mediators (39). Peripheral pathology in FMS induces small fiber neuropathy and mast cell activation, which sustain central sensitization through “peripheral-central signaling.”At the peripheral level, acupuncture activates TRPV1 and TRPV2 channels on mast cells, promoting degranulation and release of immune mediators like histamine and adenosine. These interact with receptors on nerve endings, initiating neuroimmune regulation (46).

Fatigue ranks as the second most prevalent symptom reported by patients with FMS, following pain, characterized by persistent mental fatigue and impaired physical recovery, rather than mere physical exhaustion (47). Acupuncture improves fatigue in FMS primarily by promoting mitochondrial function and improving muscle microcirculation. Animal studies have shown that acupuncture at ST36 can increase mitochondrial biosynthesis in muscle tissue by approximately 35% and improve ATP production efficiency by 40–50% (48, 49). Additionally, an animal study reported (50) that acupuncture significantly increases the levels of the antioxidant enzyme glutathione, enhances antioxidant responses, and improves muscle strength and function.

Depressive symptoms in FMS patients are not merely psychological reactions but have a clear neurobiological basis. In FMS patients, chronic stress induces significant reductions in hippocampal expression of both 5-HT and BDNF, affecting neuronal plasticity and antidepressant capacity (51, 52). The findings of this study demonstrate that acupuncture alleviates depressive symptoms in FMS patients, consistent with previous research findings. Animal experiments have shown (53, 54) that acupuncture can increase 5-HT concentrations in the hippocampus of depressed model rats while reducing monoamine oxidase activity. Additionally, studies have found (55, 56) that electroacupuncture stimulation significantly enhances BDNF levels in the hippocampus of depressed rats, activates TrkB receptors and their downstream signaling pathways, and promotes neural regeneration. These mechanisms may explain the therapeutic effects of acupuncture on depressive symptoms associated with FMS.

In subgroup meta-analysis, we primarily examined the effects of acupuncture intervention duration and different intervention measures in the control group on meta-analysis results and heterogeneity. Acupuncture intervention duration may be a source of heterogeneity in VAS, FIQ, and sleep outcomes. It had no significant impact on meta-analysis results for VAS and FIQ scores but significantly influenced meta-analysis results for sleep outcomes. At 2–4 weeks, acupuncture improved sleep in FMS patients, but at 8–12 weeks, it had no significant effect on sleep. Exploring the reasons, it may be due to the acute analgesic and relaxation-inducing effects of acupuncture, which can improve sleep in the short term, but long-term improvement in sleep is not obvious. It may be that the acupuncture points used are not changed according to the symptoms and requires deeper and sustained mechanism regulation; another possible reason is that only two studies were conducted between 8 and 12 weeks, with a small sample size, which may have introduced bias in the results. In the treatment course analysis, symptom improvement was observed between 2 and 4 weeks, and acupuncture intervention between 8 and 12 weeks may be necessary to maintain efficacy. This study demonstrates that acupuncture is effective relative to sham acupuncture and medication, though the effect sizes differ. This implies that regardless of its mechanism, acupuncture remains a valuable therapeutic option. Further investigation with larger sample sizes is warranted to validate the effects of varying acupuncture treatment durations on sleep in FMS.

In clinical practice, in addition to acupuncture treatment, the selection of acupoints also significantly influences treatment outcomes. According to a study (57), commonly used acupoints include BL18, BL20, A Shi, SP6, and LI4. Additionally, based on the different symptoms associated with FMS, different acupoints are selected. For example, for depression, GV20, GV29, and LR3 are used; for fatigue, ST36 and BL23 are used; and for sleep disorders, PC6 and BL15 are used. Therefore, in acupuncture treatment for FMS, in addition to the basic commonly used acupoints, different acupoints should be added based on the accompanying symptoms presented by the patient to enhance therapeutic efficacy. This study and previous research demonstrate that acupuncture reliably improves pain, depression, and fatigue in FMS patients. It may be considered for inclusion in clinical pathways as a complementary or alternative approach to pharmacological treatment. Given variations in treatment response across different durations, clinical adjustments based on disease severity are recommended: mild cases may undergo 2–4 weeks of treatment, while moderate to severe cases should extend therapy to 8–12 weeks to sustain efficacy. Establish interdisciplinary teams to jointly advance evidence translation and resolve professional coordination issues in clinical practice.

Among the included studies, 6 studies reported local adverse reactions to acupuncture (discomfort, pain, bruising, hematoma), 5 studiesmentioned systemic adverse reactions (dizziness, nausea, weakness, vasovagal symptoms), while the remaining studies did not report any adverse reactions. These reactions were typically transient and self-limiting, well tolerated by patients, and rarely led to treatment discontinuation. Therefore, during acupuncture treatment, attention should be paid to patients with bleeding tendencies or a history of cardiovascular or cerebrovascular disease to prevent bleeding, nausea and dizziness.

### Limitations and outlook

1. Some of the articles included in this study did not use a blind method, this may have some effect on the outcome; 2. The sample size for some of the study indicators was too small, and the results may have been affected by the small sample effect; 3. High heterogeneity across some outcomes, and some included studies have unclear or high risk of bias may introduced a certain degree of bias in the results; 4. Potential publication bias due to concentration of single-center Chinese RCTs. These findings should be verified in future higher-quality studies with larger sample sizes. At the same time, this study may provide evidence-based support for acupuncture treatment of FMS and serve as a reference for subsequent clinical studies.

## 5 Conclusions

Acupuncture can reduce patients' pain, improve depression and fatigue, and lower overall FIQ scores to treat FMS. Future high-quality research with larger sample is necessary to corroborate these results.

## Data Availability

The original contributions presented in the study are included in the article/supplementary material, further inquiries can be directed to the corresponding author.
