# Supplementary material for: The efficacy of acupuncture treatment for fibromyalgia syndrome: a systematic review and meta-analysis
Source: Front Med (Lausanne). 2026 Jan 16;12:1710642. doi: 10.3389/fmed.2025.1710642 (PMC12855461; doi:10.3389/fmed.2025.1710642)
Supplement: Supplementary file 1 [file Data_Sheet_1.pdf]

Supplementary Material

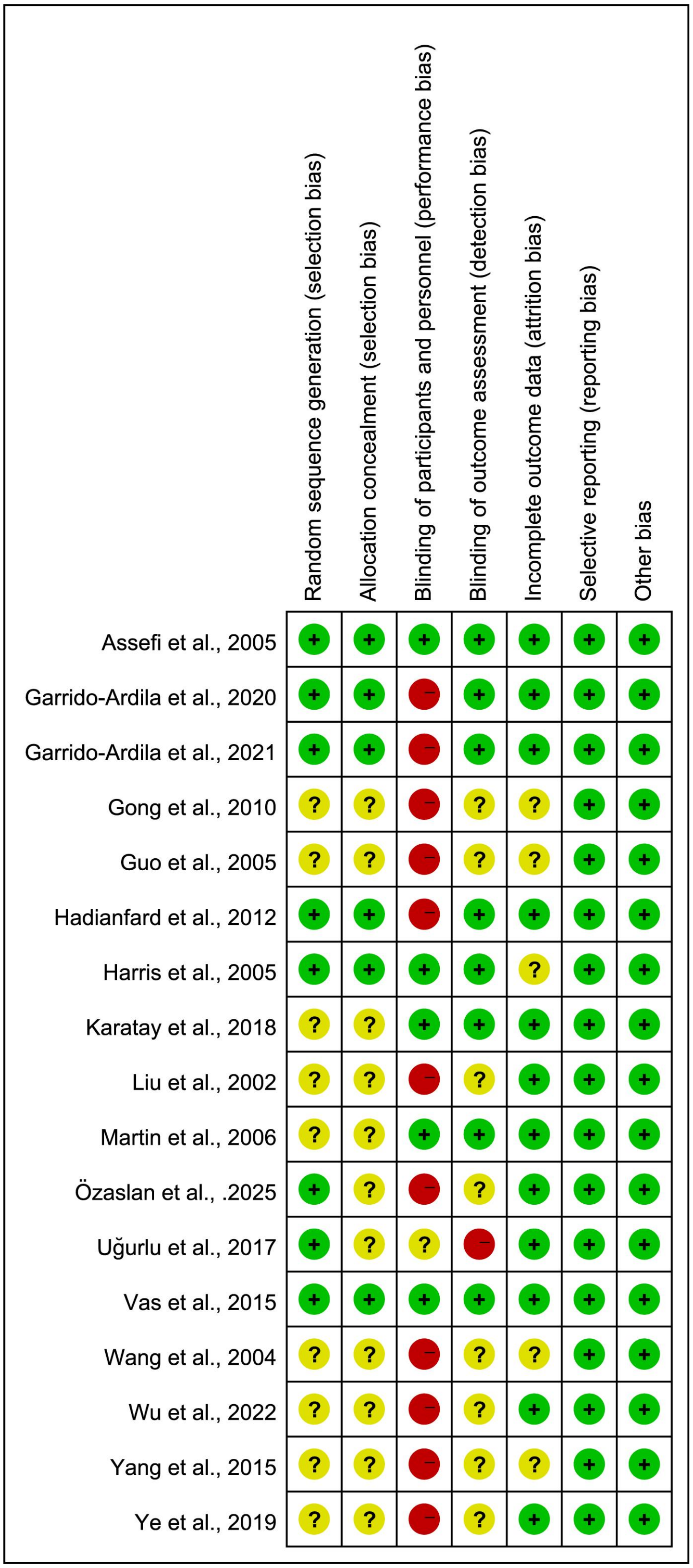

Supplementary Figure 1. Risk of bias.

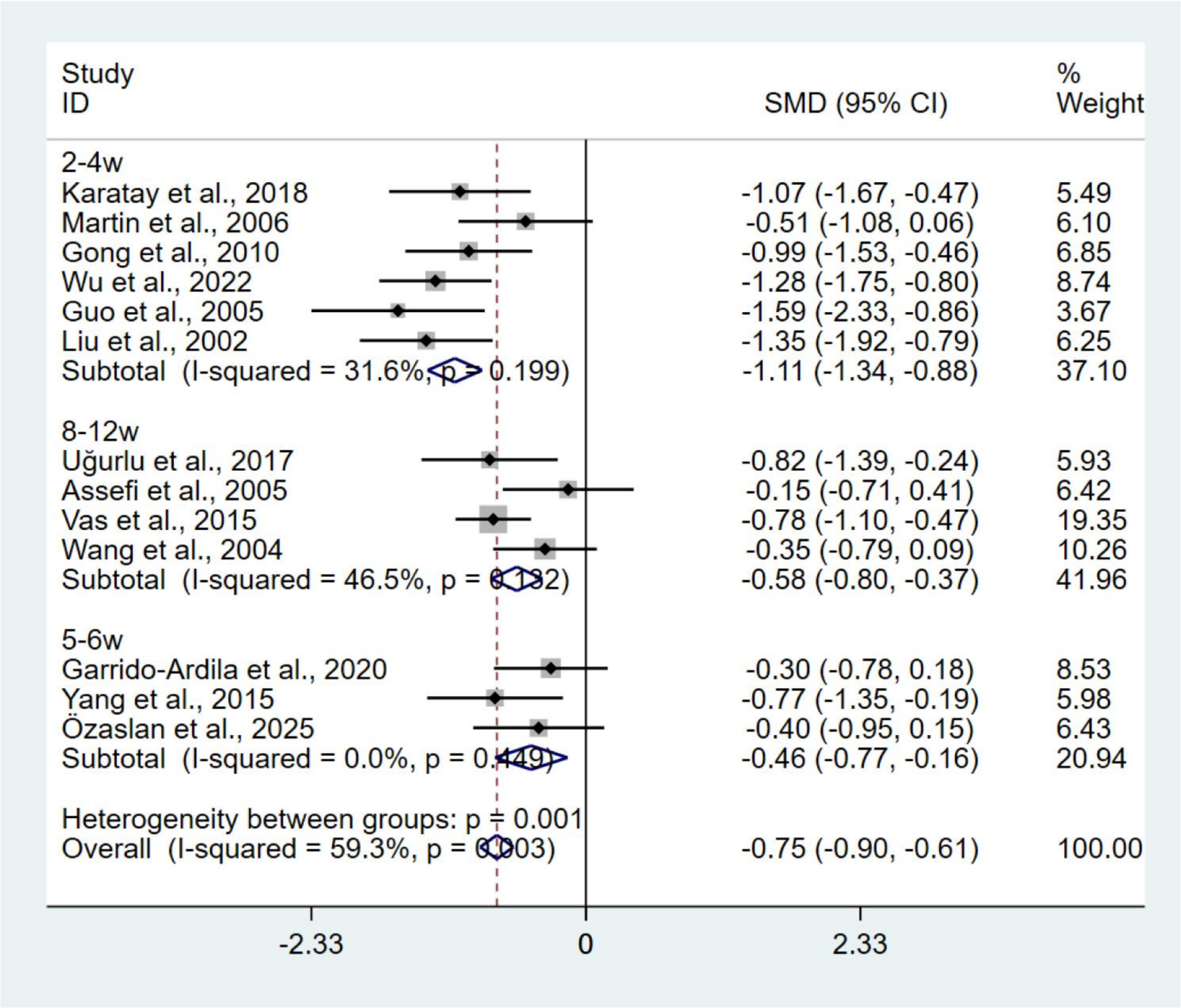

Supplementary Figure 2. Subgroup meta-analysis of VAS.

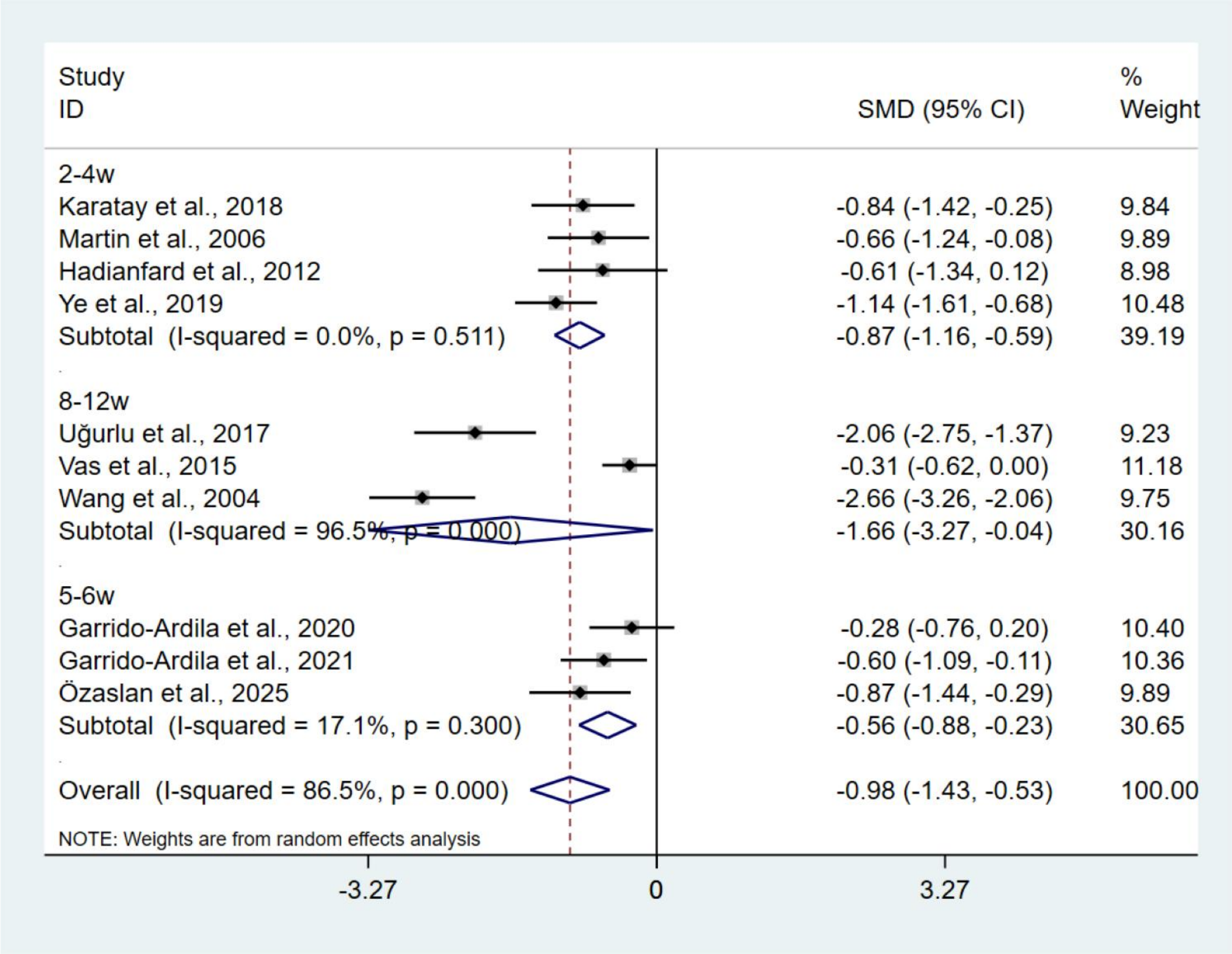

Supplementary Figure 3. Subgroup meta-analysis of FIQ.

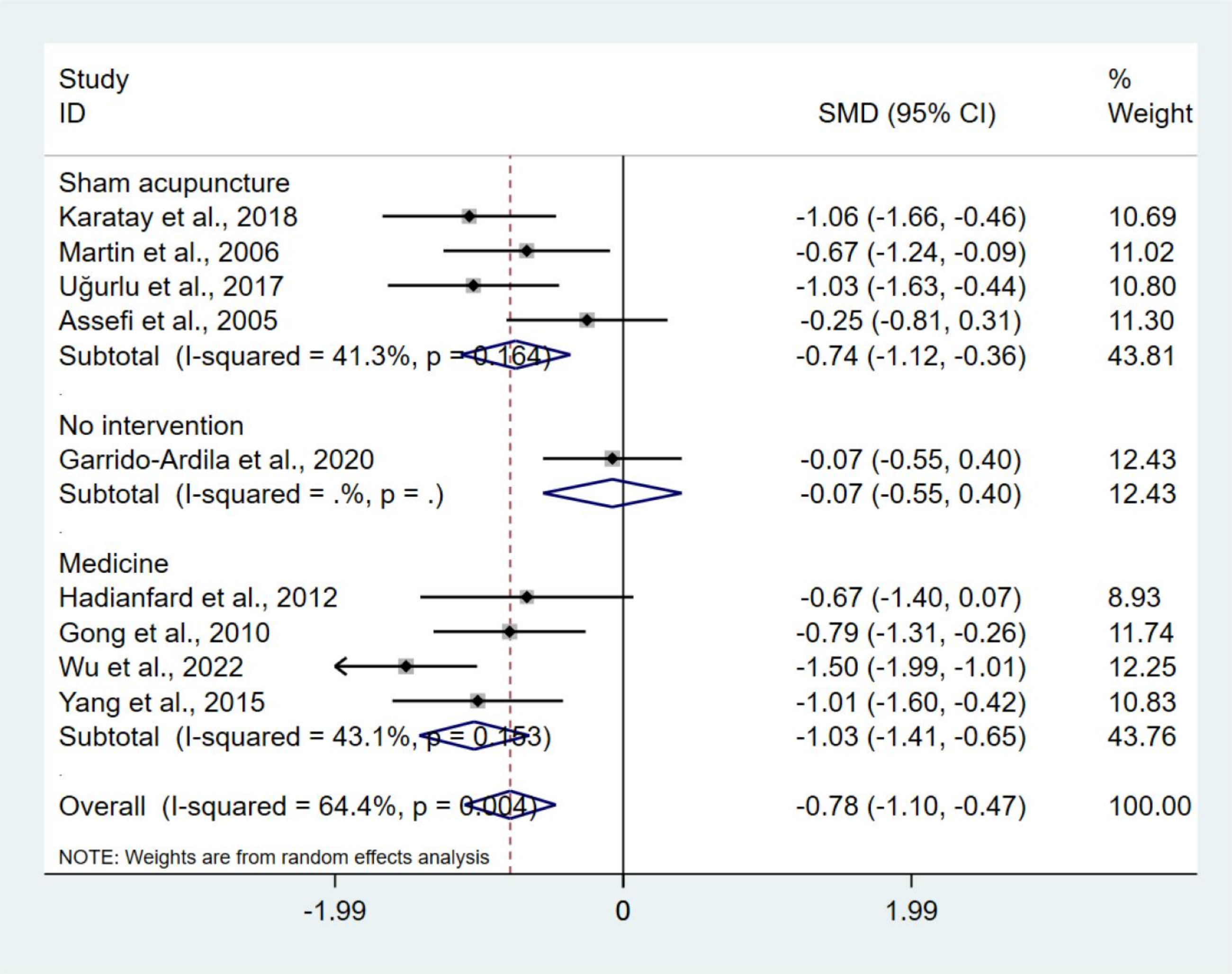

Supplementary Figure 4. Subgroup meta-analysis of depression.

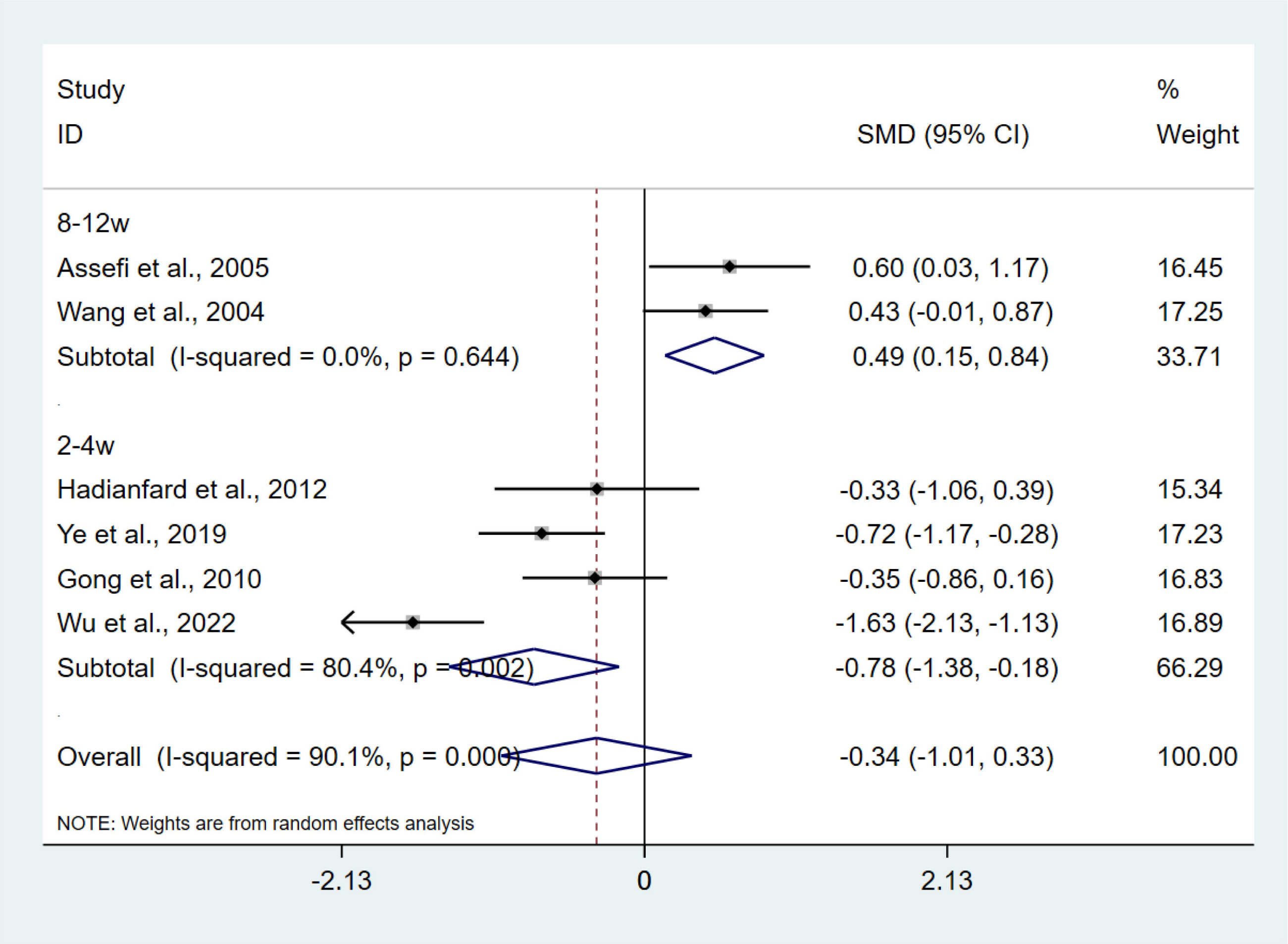

Supplementary Figure 5. Subgroup meta-analysis of sleep.

```
Meta-analysis regression                          No of studies =     13
                                                  tau^2 method        reml
                                                  tau^2 estimate =   .0502

Successive values of tau^2 differ by less than 10^-4 :convergence achieved
```

	Coef.	Std. Err.	z	P>|z|	[95% Conf.	Interval]
var3	.2899805	.1105162	2.62	0.009	.0733728	.5065882
_cons	-1.329835	.2353411	-5.65	0.000	-1.791095	-.8685754

Supplementary Figure 6. Meta-regression analysis of VAS

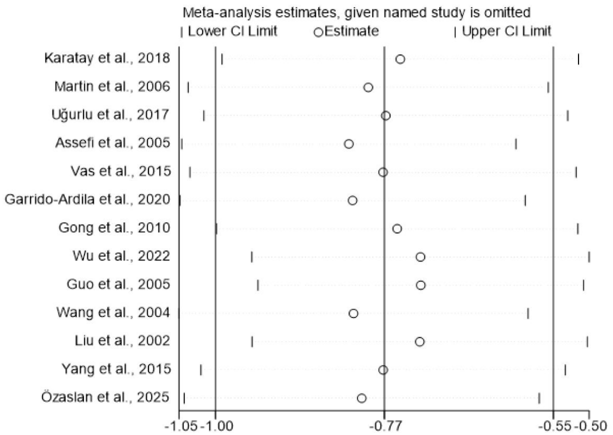

VAS

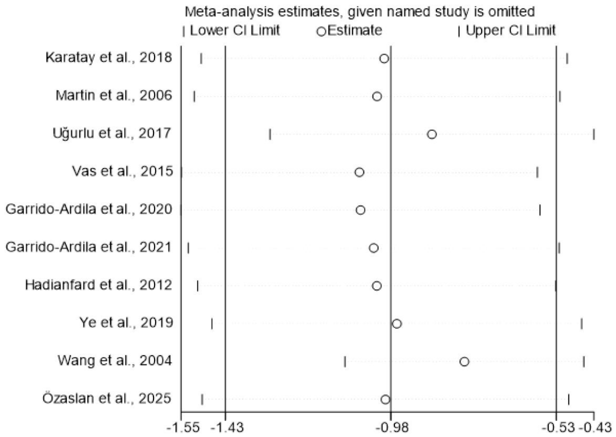

FIQ

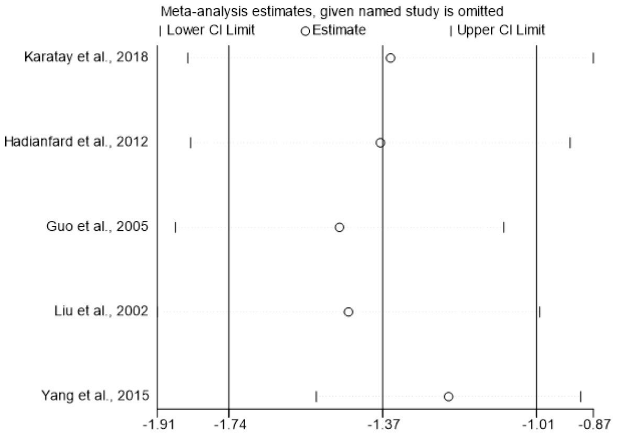

Number of pain points

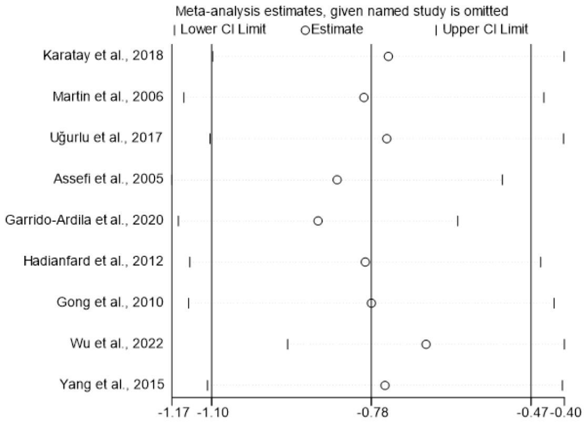

Depression

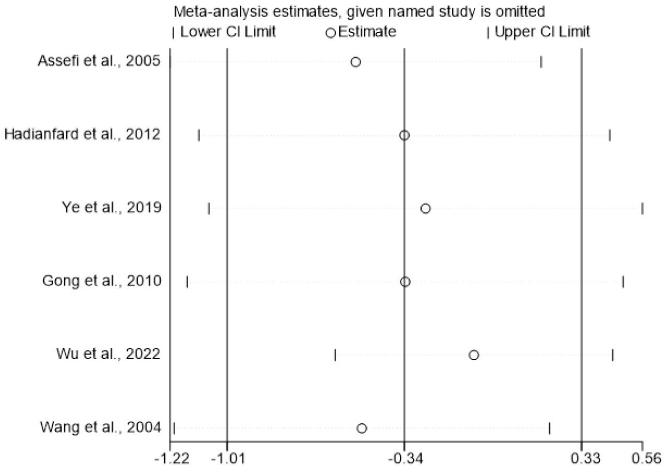

Number of pain points

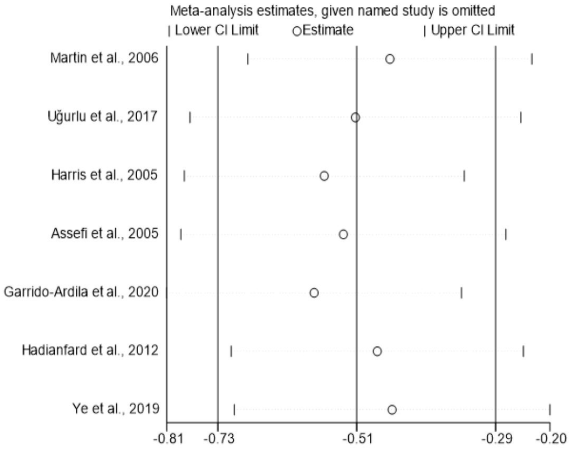

Fatigue

Supplementary Figure 7. Sensitivity analysis.

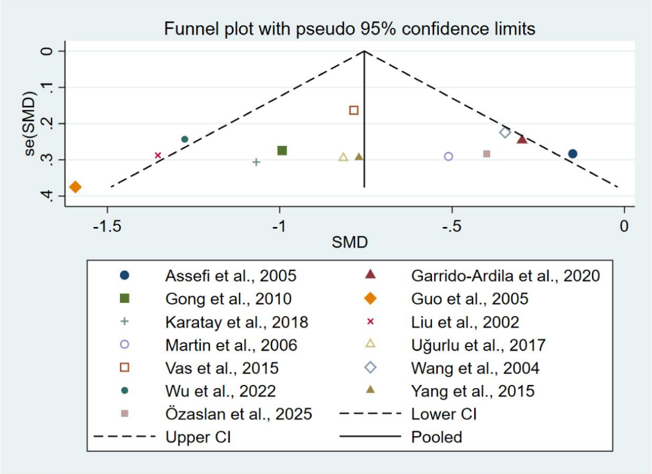

VAS

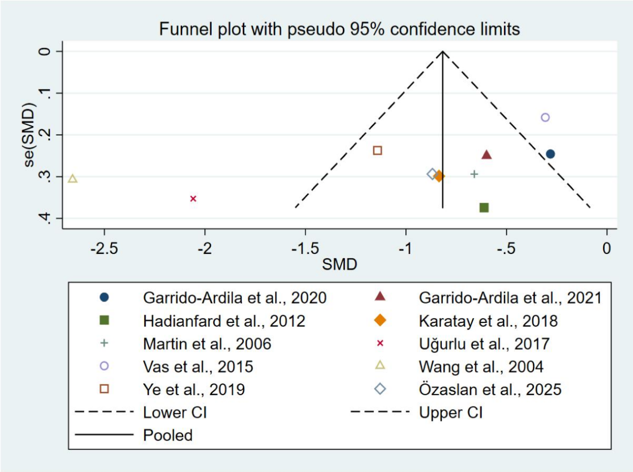

FIQ

Supplementary Figure 8. Publication bias.

Supplementary Table 1 Search strategy

Pubmed

(((((Acupuncture[MeSH Terms]) OR (Acupuncture Therapy[Title/Abstract])) OR (Acupuncture Analgesia[Title/Abstract])) OR (electroacupuncture[Title/Abstract])) OR (Acupuncture)) AND ((((((Fibromyalgia[MeSH Terms]) OR (Fibromyalgias[Title/Abstract])) OR (Fibromyalgia Syndrome[Title/Abstract])) OR (Diffuse Myofascial Pain Syndrome[Title/Abstract])) OR (Fibrositis[Title/Abstract])) OR (Fibromyalgia))

Web of Science

#1 Acupuncture (Topic) or Acupuncture Therapy (All Fields) or Acupuncture Analgesia (All Fields) or electroacupuncture (All Fields)

#2 Fibromyalgia (Topic) or Fibromyalgias (All Fields) or Fibromyalgia Syndrome (All Fields) or Diffuse Myofascial Pain Syndrome (All Fields) or Fibrositis (All Fields)

#3 #1 and #2

Embase

#1 acupuncture:ab,ti OR 'acupuncture therapy':ab,ti OR 'acupuncture analgesia':ab,ti OR electroacupuncture:ab,ti

#2 fibromyalgia:ab,ti OR fibromyalgias:ab,ti OR 'fibromyalgia syndrome':ab,ti OR 'diffuse myofascial pain syndrome':ab,ti OR fibrositis:ab,ti

#3 'clinical article'/de OR 'major clinical study'/de OR 'randomized controlled trial'/de

#4 #1 and #2 and #3

The Cochrane Library

#1 ("acupuncture"):ti,ab,kw OR ("acupuncture analgesia"):ti,ab,kw OR (AcupunctureTherapy):ti,ab,kw OR (electroacupuncture):ti,ab,kw

#2 (Fibromyalgia):ti,ab,kw OR (Fibromyalgias):ti,ab,kw OR (Fibromyalgia Syndrome):ti,ab,kw OR (Diffuse Myofascial Pain Syndrome):ti,ab,kw OR (Fibrositis):ti,ab,kw

#3 #1 and #2

CNKI

（主题：针灸 + 针灸治疗 + 针灸疗法 + 针灸临床 + 针灸方法 + 常规针灸）OR（主题：针刺 + 针刺治疗 + 针刺疗法 + 针刺法 + 针刺法治疗 + 针刺镇痛）OR（主题：电针 + 电针治疗 + 电针疗法 + 电针镇痛 + 电针穴位 + 常规电针）AND（主题：纤维肌痛 + 纤维肌痛综合征 + 纤维肌痛症 + '纤维肌痛综合征(fms)' + 纤维肌痛综合症 + 纤维肌痛患者）OR（主题：纤维肌痛 + 纤维肌痛征 + 纤维肌痛症状 + 纤维肌痛患者）

Sinomed

("纤维肌痛"[常用字段:智能] OR"纤维肌痛综合征"[常用字段:智能]) AND("针灸"[常用字段:智能] OR "针刺"[常用字段:智能]OR"电针"[常用字段:智能])

VIP

(题名或关键词=针灸 OR 题名或关键词=针刺 OR 题名或关键词=电针)AND(题名或关键词=纤维肌痛综合征 OR 题名或关键词=纤维肌痛 OR 题名或关键词=纤维肌疼痛)

WanFang

（主题:(纤维肌痛) or 主题:(纤维肌痛综合征) or 主题:(纤维肌疼痛)) AND (主题:(针灸) or 主题:(针刺) or 主题:(电针))
